# Femoral diameter and stem type are independent risk factors for ARMD in the Large-headed ASR THR group

**DOI:** 10.1186/s12891-015-0566-6

**Published:** 2015-05-15

**Authors:** Aleksi Reito, Petra Elo, Timo Puolakka, Jorma Pajamäki, Antti Eskelinen

**Affiliations:** Coxa Hospital for Joint Replacement, Biokatu 6b, 33520 Tampere, Finland

**Keywords:** Metal-on-metal, Pseudotumour, Revision, Total hip replacement, ARMD, Cox regression, Risk factors

## Abstract

**Background:**

Adverse soft-tissue reaction to metal debris (ARMD) continues to be major source of concern in metal-on-metal (MoM) hip replacements. In our earlier study we were able to establish several risk factors for ARMD in patients who had received a small-diameter (<50 mm) Articular Surface Replacement (ASR, DePuy, Warsaw, IN, USA). The aims of the present study were to analyze whether these previously established risk factors also apply to patients who have received a large-headed (>50 mm) ASR™ XL THR.

**Methods:**

Large-headed ASR total hip replacements were used in 225 operations (196 patients) at our institution. 176 patients (203 hips) attended a screening programme, consisting of a clinical evaluation, whole blood cobalt and chromium measurements, and cross-sectional imaging.

**Results:**

Revision surgery was performed on 84 hips (37%) in 75 patients. ARMD was diagnosed in the majority (n = 73 [87%]) of these revisions. Cumulative 8-year survivorship was 52%. The previously established risk factors for ARMD were not applicable. Interestingly, increasing femoral diameter and stem type were identified as independent risk factors for ARMD but reduced cup coverage had no significant association with ARMD.

**Conclusions:**

Stem type and increasing femoral size as independent risk factors for ARMD in the cohort of ASR XL THR patients, support the importance of taper failure in the development of ARMD. The present results suggest that the degree of taper failure may be variable and dependent on the taper design.

## Background

Adverse soft-tissue reaction to metal debris (ARMD) continues to be a major source of concern in metal-on-metal (MoM) hip replacements. In addition to Articular Surface Replacement (ASR™; DePuy, Warsaw, IN, USA) several other large-diameter metal-on-metal (LD-MoM) total hip replacements (THR) have been recalled along with Medical Device Alerts owing to high prevalence of ARMD [1,2]. It is well known that a major aetiological factor resulting in failure in MoM THRs is excessive wear originating from the taper surface [3-5]. According to registry studies and clinical patient series, however, MoM hip resurfacing (HR) seems to be successful only if large femoral sizes (>50 mm) are used [6-9].

In earlier study we were able to identify several risk factors for ARMD in patients who had received a small-diameter (<50 mm) ASR hip replacement [10]. Small femoral diameter alone has been associated with an increased risk of ARMD [11,12]. With increasing femoral size articular coverage increases due to larger hemisphere of the cup [13]. This further reduces the risk of edge-loading [14,15]. However, the prevalence of ARMD has been shown to be high with larger diameter (>50 mm) MoM hip replacements [3]. It is not exactly known whether this is solely due to taper wear and damage due to corrosion or if edge-loading and subsequent bearing surface wear also have an affect . Presumably both have an influence to higher failure rate. Registry studies suggest significantly higher revision rates with large-diameter (>50 mm) THR compared to large diameter (>50 mm) HRs when the same bearing couple is used [7].

The aims of our study were (1) to analyse and report the prevalence of ARMD among patients who received a large-headed (>50 mm) ASR™ XL THR system at our institution, and (2) to investigate whether previously identified risk factors apply to this specific patient population. To achieve these goals, we used data obtained from a mass screening programme implemented at our institution for these patients.

## Methods

### Screening programme

DePuy Orthopaedics voluntarily recalled their ASR™ MOM hip system in August 2010. After the UK Medicines and Healthcare Products Regulatory Agency announced a medical device alert regarding ASR™ hip arthroplasty implants in September 2010, our institution established a mass screening programme to identify possible articulation-related complications in patients who had received an ASR™ XL prosthesis during THR at our institution [1]. The screening process was described in detail in our earlier study [10]. Briefly, all patients received an OHS questionnaire, were referred to whole blood metal ion (chrome and cobalt) measurements, to plain hip radiographs as well as to cross-sectional imaging. All patients also underwent physical examination (including HHS) at our outpatient clinic.

### Study population

One thousand and thirty-six ASR™ MOM hip arthroplasties were performed on 887 patients at our institution between March 2004 and December 2009. In 554 operations (473 patients), a femoral head size greater than 50 mm was used. Of these 473 patients, 196 (225 hips) received an ASR™ XL THR prosthesis. Stems manufactured by DePuy were used in all ASR™ XL THRs: a proximally coated Summit® stem in 149 (66%), a hydroxyapatite-coated Corail® stem in 53 (24%), and an S-ROM® stem in 21 (9%) operations, respectively. Furthermore, a short Proxima™ stem was used in two operations (1%). All these stems were manufactured by the same company (DePuy). They all have identical 12/14 Morse taper made from cobalt-chrome alloy. The current literature does not describe possible differences in surface finish or roughness of taper between different stem brands. All living patients who had not had revision surgery with a femoral head size greater than 50 mm were invited to participate in a screening programme, and 176 agreed to do so. A written informed consent was obtained from all patients participating in this study. We obtained permission to perform this study from the ethics committee (Regional Ethics Committee in The Pirkanmaa Hospital District’s Science Centre) of the hospital district in which the study was conducted.

### Surgical technique

All primary operations were performed by or under the direct supervision of seven experienced hip surgeons (JP, TP, PH, PK, TM, UP, HS) and according to the standard protocol at our institution. A posterior approach was used in all cases and external rotators were detached along the incision of the posterior capsule and reattached with absorbable sutures through drill holes to the greater trochanter. Postoperatively patients were allowed immediate full weight bearing with crutches and without any major restrictions on movement.

### Revisions

Failure was defined as a revision operation secondary to an adverse reaction to metal debris. Revision surgery was considered if (1) a clear pseudotumour (Imperial class 2A,2B or 3) observed on cross-sectional imaging regardless of symptoms or whole blood metal ion levels; or (2) the patient had elevated whole blood metal ion levels and hip symptoms despite a normal finding in cross-sectional imaging; or (3) the patient had a continuously symptomatic hip or progressive symptoms regardless of imaging findings or metal ion levels. Symptoms included hip pain, discomfort, sense of instability, and/or impaired function of the hip and sounds from the hip (clacking, squeaking). Whole blood metal ion levels were regarded as being elevated if either chromium or cobalt exceeded 5 ppb. Diagnosis of adverse reactions to metal debris was based on perioperative findings. Failure was classified as being secondary to adverse reactions to metal debris if the following criteria were met: (1) there was presence of metallosis or macroscopic synovitis in the joint; and/or (2) a pseudotumor was found during revision; and/or (3) a moderate to large amount of perivascular lymphocytes along with tissue necrosis and/or fibrin deposition was seen in the histopathologic sample; and (4) perioperatively there was no evidence of component loosening or periprosthetic fracture. Furthermore, infection was ruled out by multiple (at least five) bacterial cultures obtained during revision surgery.

### Cross-sectional imaging

Of all 176 patients attending screening, 172 patients (97%) underwent cross-sectional imaging. MRI was performed on 149 patients (172 hips) and ultrasonography in the remaining 23 patients (27 hips). Two patients (two hips) died and one patient (one hip) had a late infection prior to any imaging. One patient declined to undergo any imaging. MRI was performed with two 1.5-T machines (Siemens Magnetom Avanto 1.5 T; Siemens Healthcare, Erlangen, Germany; and GE Signa HD 1.5 T; General Electric Healthcare, Waukesha, WI, USA). All examinations were done with coronal and axial T1-weighted fast spin echo and coronal, axial, and sagittal short tau inversion recovery.

MRI findings were categorized using Imperial classification [16]. In this classification type 1 PT indicates a PT with thin walls and fluid-like core signal. Class 2A indicates a PT with thick or irregular walls and a fluid-like core signal. Class 2B PT refers to PT with thick or irregular walls and atypical fluid core. Class 3 indicates a solid PT. MRI scans were analysed by a musculoskeletal radiologist (co-author PE). In the US examination, pseudotumour was defined as a cystic, thick-walled or solid extra-articular mass adjacent to the hip joint. US examinations were performed with Logiq e9 (GE Healthcare, Wisconsin, USA) and graded by the same musculoskeletal radiologist.

### Metal ion analysis

Whole blood metal ion levels were available for all patients participating in screening. The protocol for obtaining and assessing of blood samples has been described earlier [10].

### Statistics

Student’s *t*-test was used when comparing normally distributed variables between groups and variables violating this assumption were compared using the Mann-Whitney *U* test. For the purposes of the Cox regression analysis continuous variables were distributed to appropriate subgroups. Cup coverage was categorized as described earlier [10]. For age, a cutoff value of 50 years was used. A cutoff value of 40 years used by others would have resulted in too small subgroups, since only 10 patients in our study group were younger than 40 years [12]. Preoperative total ROM was divided into two groups based on the mean value minus ½ SD, which yielded the following distribution: less than 100° and 100° or greater. Age, preoperative range of motion (ROM), cup coverage, gender and diagnosis (primary osteoarthritis vs. other diagnosis), femoral diameter as continuous variable and stem type were studied as risk factors for adverse reactions to metal debris. The proportion of patients with bilateral implants was relatively high (29%). There were considerable differences in the distribution of stem concepts between THR subgroups: Summit stem was used in 63% of the operations in the unilateral THRs, compared of 73% in the bilateral THRs. The difference was almost significant (Chi-square, p = .067). If there is an underlying patient susceptibility predisposing to ARMD, patients with bilateral implants may be at elevated risk of revision of both hips and this may result in bias in the analysis due to differences in component selection. Therefore Cox regression analysis was performed twice in both implant groups: in the first stage only unilateral patients were included and in second stage all patients were included. Cox regression analysis was used to estimate the the adjusted risk ratios of the different variables on the risk of adverse reactions to metal debris-related failure. The assumption of proportional hazards was tested using scaled Schoenfeld residuals. No violations of the assumption was seen (p > .05). Cox regression analysis was performed including all variables in the analysis at the same time. The Wald test was applied to calculate p values for data obtained from the Cox multiple regression analysis. Comparison of survivorship by strata factor was performed using the log-rank test. The significance level was set at 0.05. Statistical analyses were conducted with IBM Statistics Version 19.0 (SPSS, Chicago, IL, USA) and Stata 10 (College Station, TX, USA).

## Results

The mean age of patients was 60.3 years (SD 10.3) (Table 1). Mean follow-up was 5.4 years (SD 2.1) (Table 2). Clinical outcome scores and measurements are shown in Table 2.

**Table 1:** **Demographics of the patients**

**Demographic**		
**Patients (n)**	**Hips (n)**	**196**
		**225**
**Age**	Mean (SD, range)	60.3 (10.3, 25 to 84)
	<50 years	25 (15.7%)
	≥50 years	171 (84.3%)
**Sex**	Male	181 (92.3%)
	Female	15 (7.7%)
**Diagnosis**	Primary OA	145 (64.9%)
	Other	80 (35.1%)
**Femoral diameter**	Median (range)	53 mm (51 to 61)
	51 mm	88
	53 mm	73
	55 mm	33
	57 mm	17
	59 mm	13
	>59 mm	1
**Preoperative ROM**	Mean (SD, range)	124° (22°, 10° to 276°)
	<100°	81 (43.4%)
	≥100°	131 (56.6%)
**Cup coverage**	Mean (SD, range)	31.4° (6.8°, 10.7° to 52.0°)
	<25°	34 (28.1%)
	≥25°	191 (71.9)

**Table 2:** **Clinical findings of the patients**

**Mean FU (SD, range)**	5.4 yrs (2.1, 0.2 to 8.0)
**Median HHS (range)**	94 (42 to 100)
**Median OHS (range)**	43 (12 to 48)
**Median WB Co (range)**	
Unilateral	3.7 ppb (0.5 to 9.10)
Bilateral	9.55 ppb (2.2 to 31.4)
**Median WB Cr (range)**	
Unilateral	1.80 ppb (0.5 to 9.1)
Bilateral	2.70 ppb (1.30 to 9.6)
**Cystic PTs (class 1)**	
**in MR**	34 (19.8%)
**in US**	3 (11.1%%)
**Thick-walled PTs (class 2A/2B)**	
in MR	22 (12.8%)
in US	4 (14.8%)

**Figure 1:**
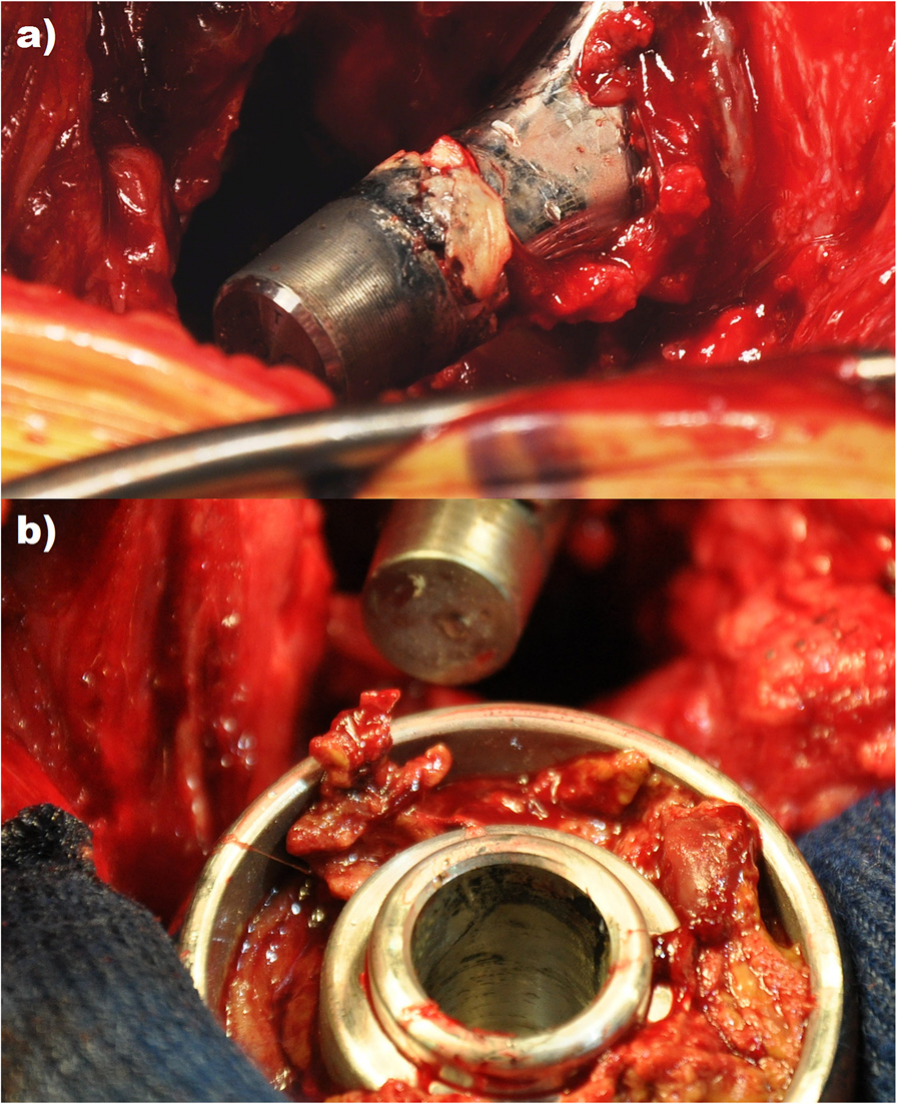
Perioperative findings in a patient undergoing revision surgery due to suspected ARMD. **A)** Male taper shows severe corrosion and fretting. **B)** Female taper shows moderate fretting and large amount of cheese-like caseotic (necrotic) tissue inside the head.

Revision surgery was performed on 84 hips in 75 patients (including those revised before the screening programme). Adverse reaction to metal debris was diagnosed in the majority (n = 73 [87%]) of these revisions (Figure 1). Of the 73 revised hips, 20 evinced a clear PT (Imperial class 2A, 2B or 3) in pre-revision imaging (18 MRI, 2 US). In 18 hips (16 MRI, 2 US) there was a cystic, thin walled PT (Imperial class 1) seen in the pre-revision imaging. In 33 hips, no PT was seen in the pre-revision imaging and in two hips no imaging was done prior to revision. In addition to 73 revisions performed for ARMD, six hips (7.1%) were revised because of deep prosthetic joint infection, two hips (2.3%) for periprosthetic fracture, two hips (2.3%) for aseptic loosening of the stem, and one hip (1.1%) for aseptic loosening of the cup.

**Figure 2:**
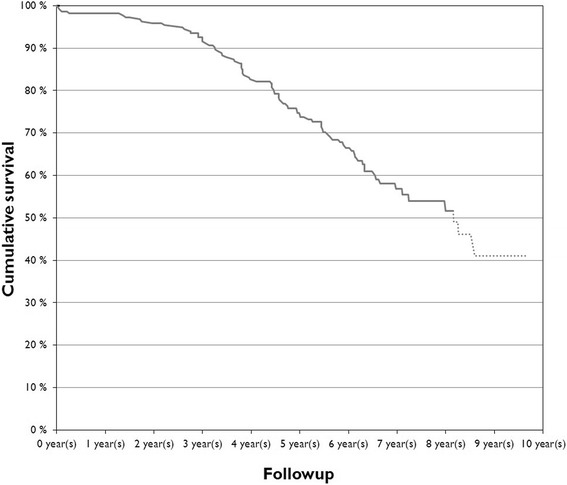
The graph shows the overall survivorship for ASR XL THR cohorts with any revision as the end point. Dotted line indicates number at risk <20.

**Figure 3:**
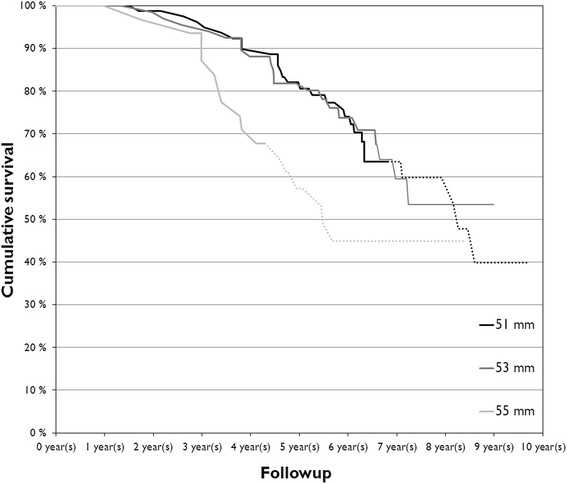
The graph shows the overall survivorship for ASR XL THR with different femoral sizes with ARMD as the end point. Dotted line indicates number at risk <20.

The prevalence of adverse reactions to metal debris was 32%. Including only unilateral patients the prevalence of ARMD was 28%. Cumulative 8-year survivorship was 52% (95% CI, 48%-56%) with any revision as the end point (Figure 2). For revision for ARMD as the end point, the cumulative 8-year survivorship was 57% (95% CI, 53%-61%). In the subgroup analyses of the THR group (only head sizes with more than 20 hips included), the poorest survivorship with ARMD as the end point was seen in hips with femoral diameter of 55 mm (p = .05) (Figure 3).

Femoral diameter and stem type were independent risk factors for ARMD in the unilateral THR group (Table 3). However, no significant risk factors were seen when bilateral cases were included (Table 3).

**Table 3:** **Results of the multivariable Cox regression analysis for the risk of ARMD in the THR cohort**

		**Unilateral THRs**		**All THRs**	
**Variable**		**HR (95% CI)**		**HR (95% CI)**	
Sex	Male	1.0 (ref)	p = .3	2.0 (ref)	p = .6
				1.26 (0.47 – 3.31)	
	Female	1.82 (0.63 – 5.21)			
Age	<50 years	1.03 (0.35 – 3.06)	p = .9	1.32 (0.56 – 3.12)	p = .5
				1.0 (ref)	
	≥50 years	1.0 (ref)			
Diagnosis	OA	1.11 (0.52 – 2.33)	p = .8	1.29 (0.68 – 2.44)	p = .4
				1.0 (ref)	
	Other	1.0 (ref)			
Cup coverage	<25 degrees	1.0 (ref)	p = .2	1.0 (ref)	p = .5
				1.28 (0.63 – 2.60)	
	≥25 degrees	1.73 (0.70 – 4.33)			
Stem	Summit	1.0 (ref)	p = .04 p = .035	2.0 (ref)	p = .061
				1.73 (0.97 – 3.06)	p = .16
	Corail	2.17 (1.03 - 4.56)		0.50 (0.19 – 1.31)	
	Other	0.26 (0.08 - 0.91)			
Femoral diameter	2 mm	1.16 (1.01 - 1.34)	p = .035	1.04 (0.94 - 1.15)	p = .5
	increment				
Preoperative ROM	<100 degrees	1.0	p = .3	1.0	p = .16
				1.46 (0.86 – 2.50)	
	≥100 degrees	1.39 (0.68 – 2.82)			

## Discussion

Small femoral size and reduced cup coverage are known risk factors for ARMD in patients with HRs but in THRs there is variation in the factors associated with ARMD (Table 4) [3,10,11,17,18]. The variation in the variables included in the analyses is large. Equal failure rates have been reported for larger diameter (>50 mm) THRs compared to small diameter (<50 mm) HRs [7] However the exact failure mechanisms have not been specifically reported in larger diameter (>50 mm) MoM hip replacements. Taper junction wear and corrosion are known to have a significant role in the failure of larger diameter THR but clinical factors related to taper failure have not been established [4,19,20]. It is not known whether previously reported risk factors apply to this subcohort. We addressed this issue using a systematic screening programme to determine the risk factors for ARMD among patients undergoing large-headed (>50 mm) ASR™ XL THRs at our institution.

**Table 4:** **Variables associated with ARMD in patients with large-diameter MoM THRs**

**Study**	**Patients (hips)**	**Statistics**	**Associated with ARMD**	**Not associated with ARMD**
Reito et al.	281 (312)	Multivariate	Reduced cup coverage	Age
				Diagnosis
			High preoperative	
			ROM	
			Corail stem	
			Female sex	
Langton et al.	418 HRs	Univariate	Small femoral diameter	Inclination angle
	87 THRs			
			High anteversion angle	
			Elevated Cr/Co levels	
Bosker et al.	119 (120)	Multivariate	Elevated Cr/Co levels	Small femoral diameter
				Anteversion angle
				Inclination angle
				Sex
				Age
Bolland et al.	185 (199)	Univariate	Elevated Co level	Cr levels
				Anteversion angle
				Inclination angle
				Femoral diameter

A limitation of our study was inadequate assessment of cup orientation. Extremes of cup version are known to be associated with an increased risk of adverse reactions to metal debris-related failure. We did not calculate cup version in this study because we lacked appropriate tools to measure version accurately. Furthermore, our survival analyses face a common orthopaedic problem since one patient may contribute two hips thus violating the assumption of independent observations [21]. We addressed this problem by analysing risk factors separately for unilateral patients and for the whole cohort. Finally, not necessarily a limitation but a matter of the validity of our results is the inclusion of variables in the risk factor analysis. Several authors have included serum or WB metal ion levels as a covariate in the regression analysis. We did not include chrome or cobalt level as covariates since we aimed at as robust a multivariable analysis as possible. Blood metal ion levels are known to correlate strongly with cup coverage and femoral diameter. Including blood metal ion levels in the analysis could possibly inflate the influence of other covariates thus falsely highlighting the importance of elevated metal ion levels alone. The main interest in the risk factor analysis was the effect of baseline variables, ie. factors which can be assessed before or during the primary operation and which are unrelated to the postoperative outcome or patient status. Including blood metal ion levels would violate this research frame. The same applies to other possible covariates such as symptoms or patient activity. Presence of symptoms as well as elevated metal ion levels has a key role in clinical decision-making and would seriously impair the power of our multivariable analysis. Moreover patient activity is likely to be affected by the presence of a painful pseudotumour. Preoperative patient activity would definitely have been an interesting covariate but our preoperative clinical assessment did not account for this and therefore we were not able to include it as a covariate.

The 8-year survival rate was 52% in this ASR XL THR group. This survival rate is comparable to the 6-year survival rate reported by Langton et al for male patients who had most likely received larger diameter components [5]. Interestingly the 7-year survival rate of 55% in the THR group in this study almost equals the survival of HR patients with femoral diameter less than 50 mm published in our earlier study and is also in accordance with that recently reported by Jack et al [7,10]. They found a significantly higher revision rate with large headed THR compared to large headed HR when an identical bearing system was used. Furthermore, no difference was seen in revision rates between large headed THR and small headed HR in that study.

Increasing femoral size and stem type were significant risk factors for ARMDIncreasing femoral size as a risk factor is a somewhat controversial finding. Larger femoral size allows better cup coverage which leads to better conformity between head and cup and a better lubrication regime [13,15]. However, hips with a femoral diameter of 55 mm may exhibit more micromotion at the taper-trunnion. Micromotion has been shown to damage the protective passivation layer of the alloy further leading to fretting and corrosion [20]. Nevertheless, our findings are in accordance with Langton et al. who reported that the relationship between the prevalence of ARMD and head size is a U-shaped curve in ASR THRs [5]. Our findings are further supported by other results by Langton et al. who showed a positive correlation between larger femoral head diameters and taper material loss [4]. Whether CoCr tapers paired with ceramic heads present with similar phenomenon warrants further investigation.

An important and novel finding in our study was also the absence of any significant effect of reduced cup coverage for the development of ARMD. This clearly emphasizes the influence of taper failure in larger-diameter hip replacements making taper failure inevitably the most important aetiological reason for the high failure prevalence. The aetiology of taper failure in larger diameter hip replacement may be purely mechanical since increased lever arm in the taper junction is associated with increased material loss in the taper [4]. Moreover, a simulator study by Panagiotidou et al. suggests that the coarser and rougher the surface finish of the taper is, the more likely is the breach of the passive film resulting in corrosion [22]. Whether the Corail stem has the coarsest and roughest surface finish thus being more vulnerable to increased lever arm in taper junction definitely warrants further research since the Corail stem is widely used with <40 mm MoM bearings and with ceramic-on-ceramic bearings.

When bilateral cases were excluded from the multivariable regression analysis stem type and increasing femoral diameter were significant independent risk factors for ARMD. However, when all cases, both unilateral and bilateral, were included no significant risk factors were identified. Conceptually the latter analysis had several violations of assumptions the most important of which is the violation of the assumption of independent observations as stated earlier. The major shift in the hazard ratios may also describe the moderation effect (interaction) between femoral head size and stem type. We assume that this interaction is mediated through the tribology of the taper. As stated earlier, tribological studies have shown that the coarser the surface, the higher the wear [22]. If the Corail stem were to have coarser surface finish, it would be more susceptible to damage and wear with increasing femoral size. Assuming that this association is absent with Summit stems including bilateral cases in the analysis would undermine the influence of the Corail stem since the majority of patients with bilateral hip replacements had Summit stems. Moreover the significance of increasing femoral head size would be lost due to over-representation of Summit stems in the study cohort.

## Conclusions

We found a high ARMD related failure rate in patients with large headed ASR XL THRs. Edge-loading due to reduced cup coverage is not an important failure mechanism in this cohort. Risk factors for ARMD in this cohort, however, suggest that taper failure is not a homogenous mechanism across different taper designs, surface finish with or without increased lever arm in the taper junction due to larger head sizes may contribute differently to taper failure. The very same cobalt-chrome and titanium tapers that have been used with large heads in MoM hips are seen in a wide range of other non-MoM THRs. Further research is warranted to find out, whether the association of taper design with taper failure is equally prominent in <40 mm MoM bearings as it is in this subcohort and whether this phenomenon goes beyond MoM hips.

## Abbreviations

ARMD: Adverse reaction to metal debris
ASR: Articular surface replacement
MoM: Metal-on-metal
PT: Pseudotumour
THR: Total hip replacement
